# Association between non-high-density lipoprotein cholesterol to high-density lipoprotein cholesterol ratio and hypertension in Chinese elderly: A cross-sectional study based on CHARLS

**DOI:** 10.1097/MD.0000000000050358

**Published:** 2026-09-04

**Authors:** Libin Huang, Shuhong Qin, Fan Qin, Yan Zhang

**Affiliations:** a Department of Neurology, Jiangbin Hospital of Guangxi Zhuang Autonomous Region, Nanning, Guangxi Zhuang Autonomous Region, China; b International Medical Department, Jiangbin Hospital of Guangxi Zhuang Autonomous Region, Nanning, Guangxi Zhuang Autonomous Region, China; c Nursing Department, Jiangbin Hospital of Guangxi Zhuang Autonomous Region, Nanning, Guangxi Zhuang Autonomous Region, China.

**Keywords:** CHARLS, elderly, hypertension, NHHR index

## Abstract

The non-high-density lipoprotein cholesterol to high-density lipoprotein cholesterol ratio (NHHR) is a novel lipid marker for cardiovascular disease. While previous studies have suggested a link between dyslipidemia and hypertension in various populations, the association between NHHR and hypertension in the older Chinese population remains unclear. This study aimed to investigate the association between NHHR and hypertension in a middle-aged and older Chinese population using data from the China Health and Retirement Longitudinal Study (CHARLS). We conducted a cross-sectional study using data from the China Health and Retirement Longitudinal Study 2015 database. NHHR was calculated as the ratio of non-high-density lipoprotein cholesterol to high-density lipoprotein cholesterol. Multivariable logistic regression models and penalized spline analysis were employed to investigate the association between NHHR and hypertension. Additionally, subgroup and sensitivity analyses were performed to assess the robustness of the findings. A total of 8342 participants (median age 67 years [interquartile range = 63–72]; 49.6% male, 50.4% female) were included in the analysis. NHHR was positively associated with prevalent hypertension. In the continuous analysis, the odds ratio for hypertension increased with each unit increment in NHHR across all multivariable-adjusted models. In the categorical model, the *P* value for the trend in the fully adjusted model was .097. A penalized spline analysis revealed a nonlinear association between NHHR and hypertension (*P* < .001). The association remained robust in subgroup and sensitivity analyses. This study found a significant association between NHHR and hypertension among older Chinese adults. The nonlinear relationship suggests a complex interplay that warrants further investigation. Prospective studies are needed to confirm these findings and clarify the temporal relationship between NHHR and hypertension.

## 1. Introduction

Hypertension is a major preventable risk factor for cardiovascular disease and mortality worldwide.^[1,2]^ Hypertension is defined as a systolic blood pressure of ≥140 mm Hg and a diastolic blood pressure of ≥90 mm Hg.^[3]^ The global prevalence of hypertension is increasing due to an aging population and increasing lifestyle risk factors, such as inadequate diets characterized by high levels of sodium and low potassium intake, along with physical inactivity.^[4]^

The World Health Organization indicates that more than 1.28 billion people aged 30 to 79 years worldwide suffer from hypertension, constituting almost 33% of the total population within this age range. Hypertension is a primary cause of early mortality worldwide. The prevalence of hypertension among the Chinese population increases with age,^[5]^ reaching a total rate of 47% among elderly hypertensive patients.^[6]^ Hypertension exerts significant stress and presents substantial obstacles to healthcare systems, particularly for poor nations. Hypertension is an important contributor to cardiovascular disease, accounting for over 10.8 million deaths globally annually.^[7]^

Studies demonstrate that patients with hypertension have a 1.35-fold higher likelihood of developing coronary heart disease, a 3.23-fold increased risk of heart failure, and are considered an essential risk factor for aortic dissection, with a risk ratio of 1.5.^[8]^ Individuals with hypertension exhibit a 3.5-fold elevated risk of cerebral hemorrhage relative to those without hypertension.^[9]^

In 2019, China linked increased systolic blood pressure to more than 25 million disability-adjusted life years due to stroke. Controlling hypertension correlates with significant decreases in cardiovascular events and mortality rates, according to considerable data.^[10]^ Therefore, the identification of novel biomarkers for evaluating hypertension risk is needed to improve prevention and treatment strategies.

Dyslipidemia is a significant risk factor for hypertension, particularly elevated low-density lipoprotein cholesterol (LDL-C), which significantly increases the risk. Oxidation of LDL-C diminishes the generation of endothelial nitric oxide synthase, thereby reducing nitric oxide production. This process leads to vasoconstriction, increased peripheral vascular resistance, and ultimately raises blood pressure.^[11]^ In parallel, hypercholesterolemia activates the renin–angiotensin system, elevating angiotensin II synthesis. This promotes vasoconstriction and consequently elevates blood pressure.^[12]^

Previous studies used lipid indicators in isolation – such as total cholesterol (TC), triglycerides (TG), LDL-C, high-density lipoprotein cholesterol (HDL-C), and non-HDL-C – to assess the risk and outcomes connected with hypertension.^[13,14]^ Studies indicate traditional lipid indicators (such as LDL-C and HDL-C) have a certain value in evaluating cardiovascular risk; nevertheless, their predictive effectiveness has limitations in hypertensive people.^[15]^

In recent years, the non-HDL-C to HDL-C ratio (NHHR) has emerged as a novel composite indication for atherogenic lipids.^[16]^ Compared with traditional lipid markers, NHHR has been demonstrated to exhibit greater effectiveness in evaluating the risk of cardiovascular and cerebrovascular diseases.^[17]^ Therefore, this study aimed to investigate the association between NHHR and hypertension in older Chinese adults using data from a large-scale nationwide survey. Elucidating this association may provide a better understanding of the lipid-related correlates of hypertension in this population.

## 2. Materials and methods

### 2.1. Ethics statement

The China Health and Retirement Longitudinal Study (CHARLS) study was reviewed and approved by the Institutional Review Board of Peking University (approval no. IRB00001052-11015), and written informed consent was obtained from all participants. The present study was a cross-sectional analysis using data from the 2015 wave of CHARLS, which included blood-based biomarkers and physical examination measurements.

### 2.2. Study population

**Figure 1.**
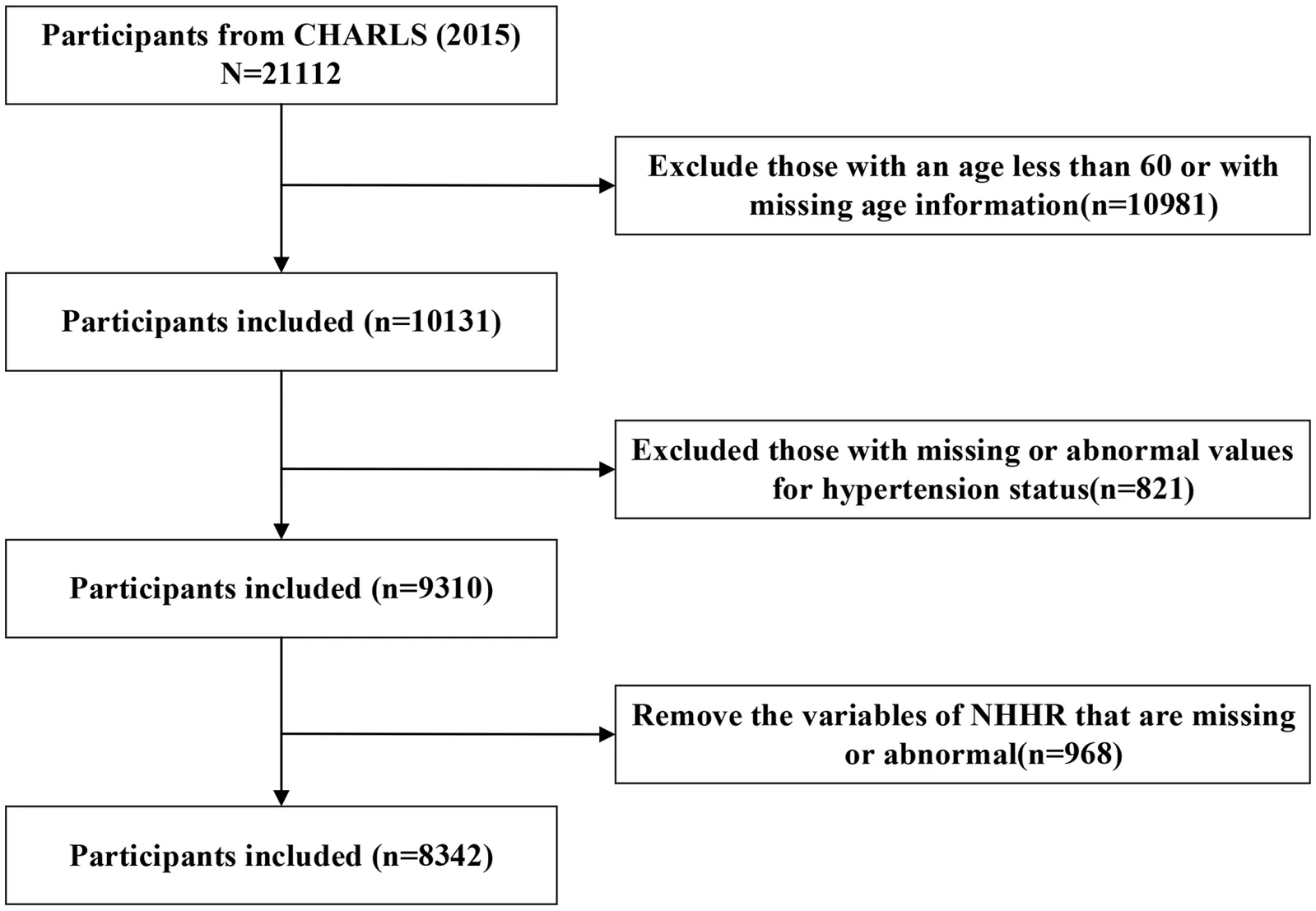
Data screening flowchart. CHARLS = China Health and Retirement Longitudinal Study.

This study used data from the 2015 wave of the CHARLS. CHARLS is a nationally representative cohort study initiated in 2011, which enrolled more than 17,000 participants aged 45 years and older at baseline. The cohort includes middle-aged and older adults from approximately 10,000 families across 450 villages, 150 counties, and 28 provinces, with follow-up surveys conducted every 2 to 3 years. Participants were interviewed in person at their residences using computer-assisted personal interviewing equipment. The survey collected demographic data, health status, medical insurance coverage, income and expenditures, physical measurements, and biochemical test results for both individuals and their households. Figure 1 shows the data screening process flowchart.

Inclusion criteria for the present analysis were people aged 60 years or older, complete data on hypertension diagnosis, and complete data on the components required to calculate NHHR (TC and HDL-C).

Exclusion criteria were missing data on hypertension diagnosis or NHHR components and poor data quality, including >40% missing data across key variables, duplicate records, or logical inconsistencies/extreme outliers that could not be reconciled.

### 2.3. Assessment of NHHR

NHHR was derived from participants’ lipid profiles and calculated using the following formulas: non-HDL-C = TC-HDL-C; NHHR = non-HDL-C/HDL-C.

### 2.4. Definition of hypertension

Hypertension was defined based on either of the following criteria: a self-reported physician diagnosis of hypertension (a positive response to the question “Have you ever been diagnosed with hypertension by a doctor?”); or self-reported current use of antihypertensive medication (a positive response to the question “Are you currently taking any treatment to control your hypertension?”).

### 2.5. Covariates

To investigate the independent association between NHHR and hypertension, we adjusted for potential confounding factors, including sociodemographic characteristics, lifestyle behaviors, and health conditions.

Sociodemographic variables included age, sex (female and male), and educational level (below primary school, primary school, junior high school, senior high school, and above). Marital status was categorized into 2 levels: married and other (unmarried, divorced, widowed). Residence type was classified as rural or urban.

Health-related variables included body mass index (BMI), diabetes, and depressive symptoms. BMI was calculated as weight in kilograms divided by height in meters squared (kg/m^2^). Diabetes was defined as a self-reported physician diagnosis. Depressive symptoms were assessed using the 10-item Center for Epidemiologic Studies Depression Scale, with a score of ≥10 indicating the presence of depressive symptoms.

### 2.6. Laboratory measurements

Blood samples were collected and processed by KingMed Diagnostics, a College of American Pathologists-accredited and International Organization for Standardization 15189-certified laboratory in China. The biomarkers assessed in this study included lipid profiles (TC, HDL-C, and LDL-C), which were measured using enzymatic methods. Detailed information on the assay methods, quality control procedures, and coefficients of variation for all biomarkers has been described previously.^[18]^

### 2.7. Statistical analysis

Data preprocessing was conducted prior to all analyses. Covariates with a missing rate of 30% or higher were excluded from the analysis. For the remaining variables with missing data, multiple imputation was performed using the Multiple Imputation by Chained Equations package to handle missing values and preserve sample size. Duplicate entries were removed, and outliers were identified and managed. Categorical variables were converted for analysis using label encoding.

Continuous variables were presented as mean ± standard deviation or median (interquartile range) for normally and non-normally distributed data, respectively. Normality was assessed using the Shapiro–Wilk test, and no transformations were applied to non-normally distributed variables. Categorical variables were presented as numbers (percentages). For intergroup comparisons, independent samples *t* tests, Mann–Whitney *U* tests, and chi-square tests (or Fisher exact tests, where appropriate) were used for normally distributed continuous, non-normally distributed continuous, and categorical variables, respectively.

NHHR was analyzed as both a continuous variable and a categorical variable (quartiles), with the lowest quartile serving as the reference group. Multivariable logistic regression models were used to examine the association between NHHR and prevalent hypertension. Three progressively adjusted models were constructed: model 1 was unadjusted; model 2 was adjusted for demographic variables (age, sex, education level, marital status, and residence); model 3 was further adjusted for BMI, smoking status, drinking status, physical activity, depression, and diabetes. Odds ratios (ORs) and 95% confidence intervals (CIs) were calculated. The *P* for trend across NHHR quartiles was calculated by modeling the median value of each quartile as a continuous variable. Multicollinearity among the independent variables in the fully adjusted model (model 3) was assessed using variance inflation factors.

To examine the dose–response relationship, a penalized spline regression analysis was performed with knots placed at the 5th, 50th, and 95th percentiles of NHHR. Subgroup analyses were conducted stratified by sex, marital status, education level, smoking status, drinking status, physical activity, depression, diabetes, and BMI to evaluate the consistency of the association.

Two sensitivity analyses were performed to assess the robustness of the findings: receiver operating characteristic curve analysis was conducted to determine an optimal cutoff value for dichotomizing NHHR. Hypertension status was used as the outcome variable, and NHHR was treated as the continuous test variable. The optimal cutoff value was selected based on the Youden index (the value maximizing sensitivity + specificity − 1); the analyses were repeated after excluding participants with hypercholesterolemia (non-HDL-C > 160 mg/dL). All statistical analyses were performed using R software (version 4.4.2; R Foundation for Statistical Computing). Two-tailed tests were used, and statistical significance was set at *P* < .05.

## 3. Results

### 3.1. Sample characteristics of the participants

Table 1 shows the demographic information of the participants. Of the 8342 participants in the final analysis, 3369 (40.38%) indicated a history of angina. The average age of participants was 67 years (range = 63–72), with 49.59% identifying as male and a median NHHR of 2.6 mg/dL (range = 2.07–3.21). NHHR was markedly elevated in hypertension patients compared with non-hypertensive patients (2.71 [2.16, 3.31] vs 2.53 [2.02, 3.12] mg/dL, *P* < .001). Notable disparities were also detected between the 2 groups for many sample variables, including sex, age, marital status, residence, smoking status, drinking status, depression, BMI, and diabetes (all *P* < .05).

**Table 1:** Characteristics of the study population.

Characteristic	Total (n = 8342)	Non-hypertension group (n = 4973)	Hypertension group (n = 3369)	*P* value
Age (yr)	67 (63, 72)	66 (62, 72)	68 (63, 73)	<.001
Sex, n (%)				<.001
Male	4137 (49.59%)	2567 (51.62%)	1570 (46.60%)	
Female	4205 (50.41%)	2406 (48.38%)	1799 (53.40%)	
Marital status, n (%)				<.001
Married	6674 (80.00%)	4062 (81.68%)	2612 (77.53%)	
Other (unmarried, divorced, widowed)	1668 (20.00%)	911 (18.32%)	757 (22.47%)	
Residence, n (%)				<.001
Village	5057 (60.62%)	3116 (62.66%)	1941 (57.61%)	
Town	3285 (39.38%)	1857 (37.34%)	1428 (42.39%)	
Education, n (%)				.840
Below primary school	4466 (53.54%)	2665 (53.59%)	1801 (53.46%)	
Primary school	2054 (24.62%)	1214 (24.41%)	840 (24.93%)	
Middle school	1171 (14.04%)	710 (14.28%)	461 (13.68%)	
High school and above	651 (7.80%)	384 (7.72%)	267 (7.93%)	
Smoking, n (%)				<.001
No	6095 (73.06%)	3457 (69.52%)	2638 (78.30%)	
Yes	2247 (26.94%)	1516 (30.48%)	731 (21.70%)	
Drinking, n (%)				<.001
No	5619 (67.36%)	3216 (64.67%)	2403 (71.33%)	
Yes	2723 (32.64%)	1757 (35.33%)	966 (28.67%)	
Exercise, n (%)				.999
No	1162 (13.93%)	693 (13.94%)	469 (13.92%)	
Yes	7180 (86.07%)	4280 (86.06%)	2900 (86.08%)	
Depressive symptoms, n (%)				<.001
No	5380 (64.49%)	3330 (66.96%)	2050 (60.85%)	
Yes	2962 (35.51%)	1643 (33.04%)	1319 (39.15%)	
Diabetes, n (%)				<.001
No	7350 (88.11%)	4613 (92.76%)	2737 (81.24%)	
Yes	992 (11.89%)	360 (7.24%)	632 (18.76%)	
BMI (kg/m^2^)	23.11 (20.77, 25.60)	23.14 (20.82, 25.72)	23.052 (20.70, 25.40)	<.001
HDL-C (mg/dL)	51 (44, 58)	51 (44, 59)	49 (42, 58)	.001
NHHR	2.6 (2.07, 3.21)	2.53 (2.02, 3.12)	2.71 (2.16, 3.31)	<.001
TC (mg/dL)	183 (161, 207)	182 (160, 206)	185 (161, 209)	.137
Waist (cm)	86 (78, 93)	84 (77, 91)	88 (81, 96)	<.001

BMI = body mass index, HDL-C = high-density lipoprotein cholesterol, NHHR = non-high-density lipoprotein cholesterol to high-density lipoprotein cholesterol ratio, Q = quartile, Ref. = reference, TC = total cholesterol.

### 3.2. The association between NHHR and hypertension

The results showed that elevated NHHR was positively associated with hypertension. In the continuous analysis, each unit increase in NHHR was significantly associated with higher odds of hypertension across all adjusted models (model 1: OR = 1.20, 95% CI = 1.1–1.26, *P* < .001; model 2: OR = 1.19, 95% CI = 1.14–1.25, *P* < .001; model 3: OR = 1.15, 95% CI = 1.10–1.21, *P* < .001).

In the categorical analysis, NHHR was divided into quartiles, with the lowest quartile (Q1) serving as the reference group. A gradually increasing odds of hypertension was observed with higher NHHR quartiles. In model 1 and model 2, the ORs for the highest quartile (Q4) were 1.66 (95% CI = 1.47–1.88) and 1.63 (95% CI = 1.44–1.85), respectively, with corresponding *P* for trend values of .031 and .043. However, after further adjustment in model 3, the association was attenuated, and the *P* for trend was .097, indicating no statistically significant linear trend (Table 2). The variance inflation factor values for all variables in model 3 ranged from 1.01 to 1.58, indicating no significant multicollinearity (Table S1, Supplemental Digital Content 1).

**Table 2:** Association between NHHR and hypertension among older Chinese adults.

Outcomes	Continuous models		Categorical model				
	OR (95% CI)	*P* value	Q1 (lowest)	Q2	Q3	Q4 (highest)	*P* for trend
Model 1	1.20 (1.14–1.26	<.001	1 (Ref.)	1.15 (1.01–1.30)	1.34 (1.19–1.52)	1.66 (1.47–1.88)	.031
Model 2	1.19 (1.14–1.25)	<.001	1 (Ref.)	1.14 (1.00–1.30)	1.32 (1.17–1.50)	1.63 (1.44–1.85)	.043
Model 3	1.15 (1.10–1.21)	<.001	1 (Ref.)	1.12 (0.98–1.27)	1.28 (1.12–1.45)	1.51 (1.33–1.72	.097

Exposure: NHHR. Outcome: hypertension.

*P* for trend: Calculated by modeling the median value of each NHHR quartile as a continuous variable to test for a linear trend across quartiles.

Model 1: unadjusted. Model 2: adjusted for demographic variables (age, sex, education level, marital status, and residence). Model 3: further adjusted for BMI, smoking status, drinking status, physical activity, depression, and diabetes.

BMI = body mass index, CI = confidence interval, NHHR = non-high-density lipoprotein cholesterol to high-density lipoprotein cholesterol ratio, OR = odds ratio, Q = quartile, Ref. = reference.

**Figure 2.**
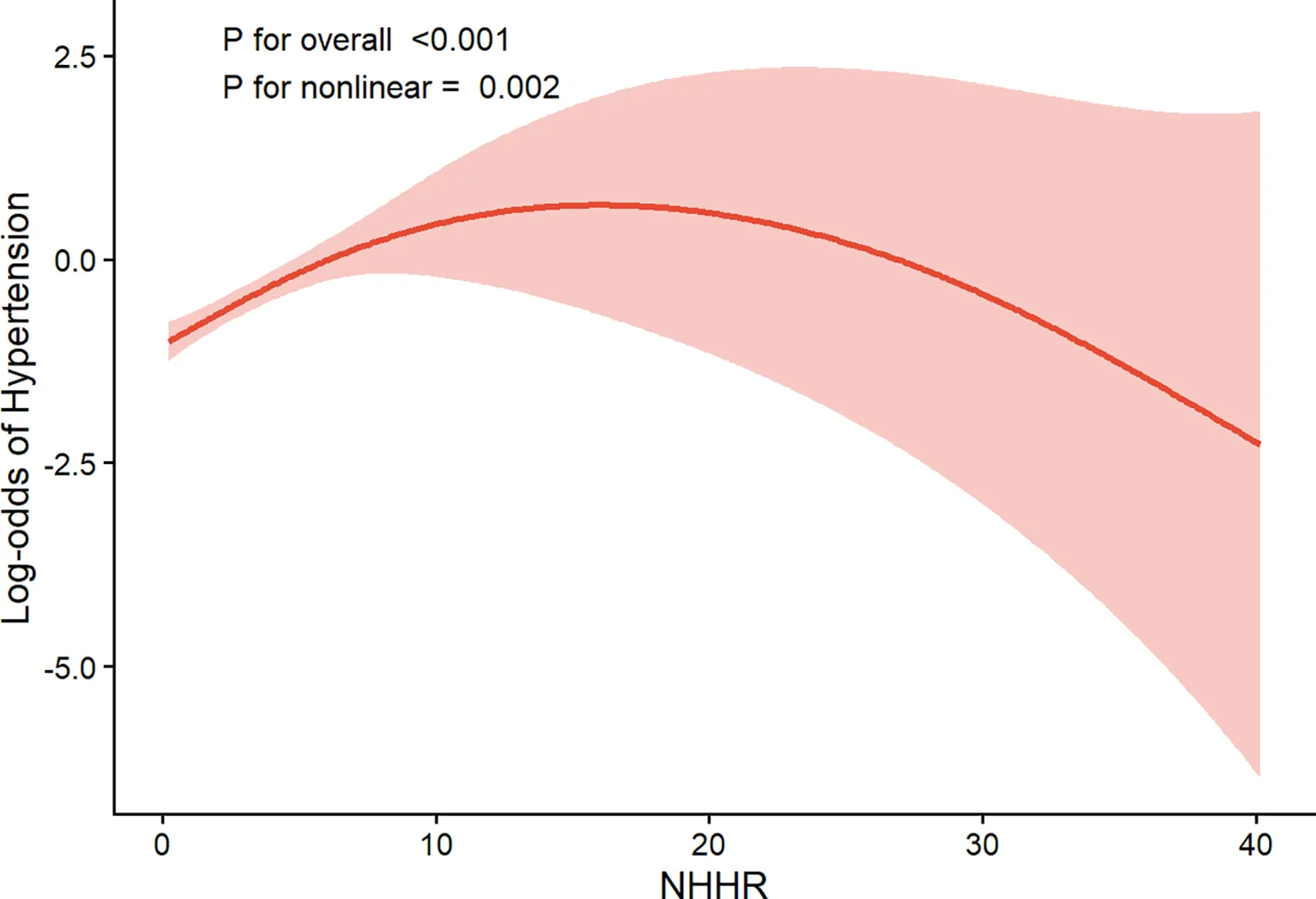
Penalized spline model of the association between NHHR and hypertension. NHHR = non-high-density lipoprotein cholesterol to high-density lipoprotein cholesterol ratio.

A penalized spline analysis revealed a nonlinear association between NHHR and hypertension (*P* for nonlinearity < .001), suggesting that the relationship between NHHR and hypertension may vary across different levels of NHHR (Fig. 2).

### 3.3. Subgroup and sensitivity analyses

Subgroup analyses were conducted to evaluate the consistency of the association between NHHR and prevalent hypertension across different populations (Table 3). No significant interactions were observed for most stratifying variables, including age, sex, BMI, marital status, residence, alcohol intake, and physical activity (all *P* for interaction > .05). However, significant interactions were detected for smoking status, diabetes, and depression (*P* for interaction < .05), suggesting that these factors may modify the association between NHHR and hypertension.

**Table 3:** Association between NHHR and hypertension in subgroups.

Subgroup	OR (95% CI)	*P* value	*P* for interaction
Sex			.831
Male	1.19 (1.11–1.28)	<.001	
Female	1.21 (1.12–1.30)	<.001	
Age (yr)			.267
60–69	1.18 (1.11–1.26)	<.001	
70–79	1.25 (1.14–1.37)	<.001	
≥80	1.37 (1.11–1.68)	.003	
BMI (kg/m^2^)			.447
<25	1.20 (1.13–1.27)	<.001	
25–30	1.25 (1.13–1.39)	<.001	
>30	1.10 (0.88–1.38)	.381	
Marital status			.885
Married	1.21 (1.14–1.28)	<.001	
Other (unmarried, divorced, widowed)	1.22 (1.09–1.37)	<.001	
Residence			.083
Village	1.16 (1.09–1.23)	<.001	
Town	1.27 (1.17–1.37)	<.001	
Smoking			.03
No	1.24 (1.17–1.32)	<.001	
Yes	1.11 (1.02–1.20)	.018	
Drinking			.427
No	1.21 (1.14–1.29)	<.001	
Yes	1.17 (1.07–1.27)	<.001	
Exercise			.482
No	1.16 (1.02–1.32)	.026	
Yes	1.22 (1.15–1.28)	<.001	
Depressive symptoms			.028
No	1.27 (1.19–1.35)	<.001	
Yes	1.13 (1.05–1.22)	.002	
Diabetes			.002
No	1.20 (1.14–1.27)	<.001	
Yes	1.02 (0.93–1.11)	.699	

All estimates were derived from multivariable logistic regression models adjusted for age, sex, BMI, marital status, residence, smoking, drinking, physical activity, depressive symptoms, and diabetes, except for the stratification variable itself.

BMI = body mass index, CI = confidence interval, NHHR = non-high-density lipoprotein cholesterol to high-density lipoprotein cholesterol ratio, OR = odds ratio.

Sensitivity analyses were performed to assess the robustness of the findings (Table 4). After excluding participants with hypercholesterolemia (non-HDL-C > 160mg/dL), the positive association between NHHR and hypertension remained significant. In the continuous analysis, each unit increase in NHHR was associated with 18% higher odds of hypertension (OR = 1.18, 95% CI = 1.10–1.27, *P* < .001), with a significant dose–response trend across quartiles (*P* for trend < .001). Compared with the lowest quartile (Q1), the ORs for Q2, Q3, and Q4 were 1.01 (95% CI = 0.87–1.16), 1.13 (95% CI = 0.98–1.31), and 1.34 (95% CI = 1.16–1.55), respectively, with Q4 reaching statistical significance (*P* < .001).

**Table 4:** Sensitivity analyses for the association between NHHR and hypertension.

Model	OR (95% CI)	*P* value	*P* for trend
Sensitivity analyses1: excluding participants with hypercholesteremia (non-HDL-C > 160 mg/dL)			<.001
Continuous model	1.18 (1.10–1.27)	<.001	
Categorical model			
Q1 (lowest)	1 (Ref.)		
Q2	1.01 (0.87–1.16)	.936	
Q3	1.13 (0.98–1.31)	.088	
Q4 (highest)	1.34 (1.16–1.55)	<.001	
Sensitivity analyses 2: NHHR as a binary variable			
≤2.63	1 (Ref.)		
>2.63	1.27 (1.15–1.41)	<.001	

The cutoff value for dichotomizing NHHR (2.63) was determined by receiver operating characteristic curve analysis based on the Youden index.

CI = confidence interval, NHHR = non-high-density lipoprotein cholesterol to high-density lipoprotein cholesterol ratio, non-HDL-C = non-high-density lipoprotein cholesterol, OR = odds ratio, Q = quartile, Ref. = reference.

When NHHR was dichotomized at the optimal cutoff value of 2.63, participants with NHHR >2.63 had 27% higher odds of hypertension compared with those with NHHR ≤2.63 (OR = 1.27, 95% CI = 1.15–1.41, *P* < .001).

## 4. Discussion

This study investigated the association between NHHR and hypertension in a nationally representative sample of older Chinese adults. After adjusting for potential confounders, NHHR was positively associated with hypertension in the continuous analysis. In the categorical analysis, a positive trend across NHHR quartiles was observed in model 1 and model 2, although the trend did not reach statistical significance in the fully adjusted model (model 3: *P* for trend = .097). A nonlinear association between NHHR and hypertension was identified using penalized spline analysis (*P* for nonlinearity < .001), suggesting that the relationship may vary across different levels of NHHR. Subgroup analyses revealed that the positive association between NHHR and hypertension was consistent across most strata, with no significant interactions observed for age, sex, BMI, marital status, residence, alcohol intake, or physical activity. However, significant interactions were detected for smoking status, diabetes, and depression, indicating that these factors may modify the association. Sensitivity analyses confirmed the robustness of the findings. After excluding participants with hypercholesterolemia, the positive association between NHHR and hypertension remained significant. Similarly, when NHHR was dichotomized at the optimal cutoff value of 2.63, participants with elevated NHHR had significantly higher odds of hypertension.

This study serves as a preliminary investigation into the association between NHHR and hypertension. Accumulating evidence suggests that NHHR is a useful biomarker for lipid-related disorders.^[19–21]^ A cross-sectional study in US adults reported a positive association between NHHR and hypertension, with each unit increase in NHHR corresponding to 60% higher odds of hypertension.^[22]^ Furthermore, numerous studies have examined the relationship between hypertension and various lipid parameters. One cross-sectional study found significantly lower serum HDL-C levels in individuals with hypertension, with HDL-C decreasing by approximately 2.3 to 2.4 mg/dL from normal blood pressure to stage 2 hypertension.^[23]^ A prospective study demonstrated that participants in the high TG/HDL-C group (≥1.97) had a 51% higher risk of developing new-onset hypertension over 7 years compared with those in the low TG/HDL-C group (<1.97).^[24]^ Other research has shown that elevated TG and LDL-C are associated with hypertension severity, while higher HDL-C levels are associated with less severe hypertension.^[13]^ Our findings are consistent with previous studies, further supporting a positive association between NHHR and hypertension. However, some studies have reported conflicting results. For example, a cross-sectional study of 62,957 Chinese men found no significant association between HDL-C or triglyceride levels and hypertension.^[14]^ These discrepancies may be attributed to differences in study populations, confounding factors, and definitions of hypertension. By utilizing a novel lipid metric, our study provides new insights into the relationship between lipid metabolism and hypertension. NHHR is an integrated indicator that reflects the balance between pro-atherogenic and anti-atherogenic lipoproteins and has been suggested to outperform traditional lipid measures in assessing atherosclerosis severity.^[16,25]^ This metric has also demonstrated good predictive performance for diabetes progression in previous studies.^[26]^ Moreover, NHHR is a noninvasive, accessible, and cost-effective measure, highlighting its potential utility in clinical practice.

The relationship between NHHR and hypertension is not fully understood, but several mechanisms may explain the association between lipid metabolism and hypertension. First, NHHR reflects the balance between pro-atherogenic and anti-atherogenic lipoprotein particles. An elevated NHHR indicates a shift toward smaller, denser low-density lipoprotein particles, which promote atherosclerotic plaque formation. Increased NHHR may result from higher non-HDL-C levels and lower HDL-C levels. Non-HDL-C particles activate the NLRP3 inflammasome, leading to the release of pro-inflammatory cytokines such as interleukin-6 and tumor necrosis factor-alpha, while reducing the anti-inflammatory buffering capacity of HDL-C. This contributes to chronic vascular inflammation and increased peripheral resistance, representing potential pathophysiological pathways linking dyslipidemia to hypertension development, as summarized in recent reviews.^[27–29]^ Second, NHHR may contribute to vascular atherosclerosis by promoting vascular inflammation, impairing endothelial function, accelerating vascular lesion progression, facilitating thrombosis, and reducing plaque stability, as demonstrated in clinical studies.^[30,31]^ Experimental studies have shown that dyslipidemia can influence hypertension through the renin–angiotensin–aldosterone system using animal and cellular models.^[32,33]^ Oxidized low-density lipoprotein increases local oxidative stress and inflammation, promoting foam cell formation and elevating angiotensin II levels. This enhances vasoconstriction, stimulates aldosterone secretion, increases sodium retention, and raises peripheral vascular resistance, as summarized in a recent review.^[34]^ Therefore, NHHR, as a composite metric of lipid metabolism disorders, may simultaneously reflect the burden of pro-atherogenic lipoproteins and the level of anti-atherogenic lipoproteins. This approach provides a more comprehensive assessment of the overall impact of lipid abnormalities on hypertension than any single lipid marker alone.

This study has several strengths. First, it used data from the CHARLS database, a nationally representative survey with a large sample size and standardized data collection. Second, we adjusted for multiple potential confounders selected based on prior literature, enhancing the reliability of the findings. Several limitations should also be acknowledged. First, hypertension status was based on self-reported diagnosis and measured blood pressure, which may introduce bias, although status was reassessed in each wave. Second, due to a lack of clinical data, we could not classify hypertension by stage or severity. Third, comorbidities such as diabetes and depression were self-reported, which may lead to residual confounding. Fourth, the study was restricted to Chinese adults aged ≥60 years, limiting generalizability. Fifth, due to the cross-sectional design, this study can only demonstrate an association between NHHR and hypertension, not a causal relationship.

## 5. Conclusion

In this cross-sectional study of older Chinese adults, elevated NHHR was positively associated with prevalent hypertension. A nonlinear relationship was also observed, suggesting that the association may vary across different levels of NHHR. However, due to the cross-sectional design, causal relationships cannot be established. Prospective cohort studies are warranted to confirm these findings and to elucidate the temporal relationship between NHHR and hypertension.

## Acknowledgments

The authors gratefully acknowledge the China Health and Retirement Longitudinal Study (CHARLS) team for providing the data support.

## Author contributions

**Conceptualization:** Libin Huang, Shuhong Qin, Fan Qin, Yan Zhang.

**Methodology:** Libin Huang, Shuhong Qin, Fan Qin.

**Software:** Libin Huang, Shuhong Qin.

**Supervision:** Yan Zhang.

**Writing – original draft:** Libin Huang, Shuhong Qin.

**Writing – review & editing:** Libin Huang, Shuhong Qin, Yan Zhang.


