# Supplementary material for: Association between non-high-density lipoprotein cholesterol to high-density lipoprotein cholesterol ratio and hypertension in Chinese elderly: A cross-sectional study based on CHARLS
Source: Medicine (Baltimore). 2026 Sep 4;105(36):e50358. doi: 10.1097/MD.0000000000050358 (PMC13549602; doi:10.1097/MD.0000000000050358)
Supplement: Supplementary file 1 [file medi-105-e50358-s001.docx]

**Supplementary Table S1 Variance inflation factors for variables in the fully adjusted model**

| Variable | VIF |
| --- | --- |
| NHHR | 1.02 |
| Age | 1.13 |
| Sex | 1.58 |
| Education | 1.25 |
| Marital status | 1.15 |
| Residence | 1.13 |
| BMI | 1.02 |
| Smoking | 1.28 |
| Drinking | 1.20 |
| Exercise | 1.01 |
| Depressive symptoms | 1.07 |
| Diabetes | 1.02 |

Note: VIF, variance inflation factor. All VIF values < 5 indicate no significant multicollinearity.
